# Molecular Dynamics Simulation and Characterization of GelMA/HA Composite Microneedles for Potential Growth Hormone Transdermal Delivery

**DOI:** 10.3390/gels12080666

**Published:** 2026-07-24

**Authors:** Jianyang Gu, Hao Zhang, Han Liu, Binghuai Peng, Yongbo Qiao, Ping Gong

**Affiliations:** 1 School of Life Science and Technology, Changchun University of Science and Technology, Changchun 130022, China; 2 Changchun Institute of Biological Products Co., Ltd., Changchun 130012, China

**Keywords:** microneedles, GelMA, hyaluronic acid, growth hormone, hGH-Fc, molecular dynamics simulation, transdermal delivery

## Abstract

To overcome the limitations of repeated GH injection therapy, gelatin methacryloyl (GelMA)/hyaluronic acid (HA) composite hydrogel microneedles were fabricated for potential non-invasive transdermal delivery of recombinant human growth hormone (rhGH) and long-acting human growth hormone Fc fusion protein (hGH-Fc). All-atom molecular dynamics simulations combined with morphological, mechanical, and scanning electron microscopy (SEM) characterization were applied to screen optimal microneedle formulations, with Fourier transform infrared spectroscopy (FTIR) analysis and Parafilm M penetration tests verifying physicochemical compatibility and insertion capability. MD simulations revealed that rhGH stabilized the matrix via hydrogen bonds and electrostatic interactions, while hGH-Fc bound mainly through van der Waals forces. Through orthogonal screening of GelMA (5%, 10%, 15%) and HA (0.5%, 1%, 2%, 5%), the 10% GelMA with 0.5–2% HA was identified as the optimal range; the representative 10% GelMA/1% HA formulation exhibited a single-tip force exceeding 1.40 N and ~500 μm penetration in Parafilm M. This study offers a theoretical and technical basis for the design and optimization of hydrogel microneedle carriers for short- and long-acting GH delivery.

## 1. Introduction

Growth hormone (GH) dominates human growth, metabolism, and tissue repair, serving as a first-line drug for clinical growth hormone deficiency treatment. Short-acting recombinant human GH (rhGH) exerts rapid therapeutic efficacy but suffers from a short circulating half-life, while Fc-modified hGH-Fc fusion protein is engineered to prolong in vivo retention for long-term treatment [1]. Nevertheless, all clinically available hGH formulations still depend on subcutaneous injection, which causes unavoidable local skin irritation and fluctuating blood drug levels. Therefore, developing minimally invasive transdermal GH delivery systems is highly desirable to overcome injection-associated drawbacks [2,3].

Microneedles can break through skin stratum corneum barriers to deliver therapeutic drugs with minimal invasion [4]. Hydrogel microneedles prepared from natural biopolymers possess superior biocompatibility, biodegradability, favorable moldability, and rapid drug-release characteristics, serving as ideal delivery carriers for protein and peptide biomacromolecules [5]. Among them, methacrylated gelatin (GelMA)/hyaluronic acid (HA) composite hydrogel microneedles combine the high protein compatibility of photocrosslinkable GelMA and favorable pore structure as well as the mechanical flexibility of HA [6,7,8,9]. Relevant studies have fabricated bilayer GelMA/HA microneedles for responsive vitamin D3 delivery, which can treat atopic dermatitis via synergistic material advantages including enhanced skin penetration and skin moisturization [10].

Although microneedles circumvent injection-induced adverse reactions, they still face inherent transdermal delivery barriers for GH administration, including protein conformational denaturation under an acidic skin microenvironment, rapid protein degradation caused by epidermal proteases, inadequate skin retention time, and limited drug loading capacity [11,12,13]. Up to now, two representative formulations of dissolvable microneedles have been developed for GH transdermal delivery with promising outcomes. Silk-based microneedles are capable of realizing sustained rhGH release and improving patient compliance, while carboxymethylcellulose/trehalose microneedles can well maintain GH bioactivity and achieve pharmacokinetic profiles comparable to conventional subcutaneous injection [14,15]. Nevertheless, neither of these two reported GH microneedle platforms has performed parallel comparative research between short-acting rhGH and long-acting hGH-Fc. More importantly, all existing GH microneedle studies merely focus on macroscopic performance evaluation, with no in-depth atomic-scale exploration of interfacial interaction differences between the two GH variants and hydrogel matrices.

A clear understanding of the molecular structural characteristics and binding behaviors of rhGH and hGH-Fc within GelMA/HA hydrogel networks is conducive to the targeted optimization of hydrogel carriers. Molecular dynamics simulation enables the atomic-level observation of protein structural variations and non-covalent intermolecular interactions, which facilitates the exploration of inherent interaction rules between proteins and hydrogel matrices. In this study, we adopted 100 ns all-atom molecular dynamics simulations to compare the differences between the two GH variants; we also optimized GelMA/HA microneedle formulations through macroscopic morphology, performed mechanical property and microscopic structure tests, verified drug–hydrogel compatibility via FTIR analysis, and assessed microneedle skin penetration capacity. This study offers fundamental theoretical and experimental support for the rational design of GH transdermal microneedle delivery systems.

## 2. Results and Discussion

### 2.1. Molecular Dynamics Simulation Results

#### 2.1.1. Conformational Distribution Characteristics

**Figure 1:**
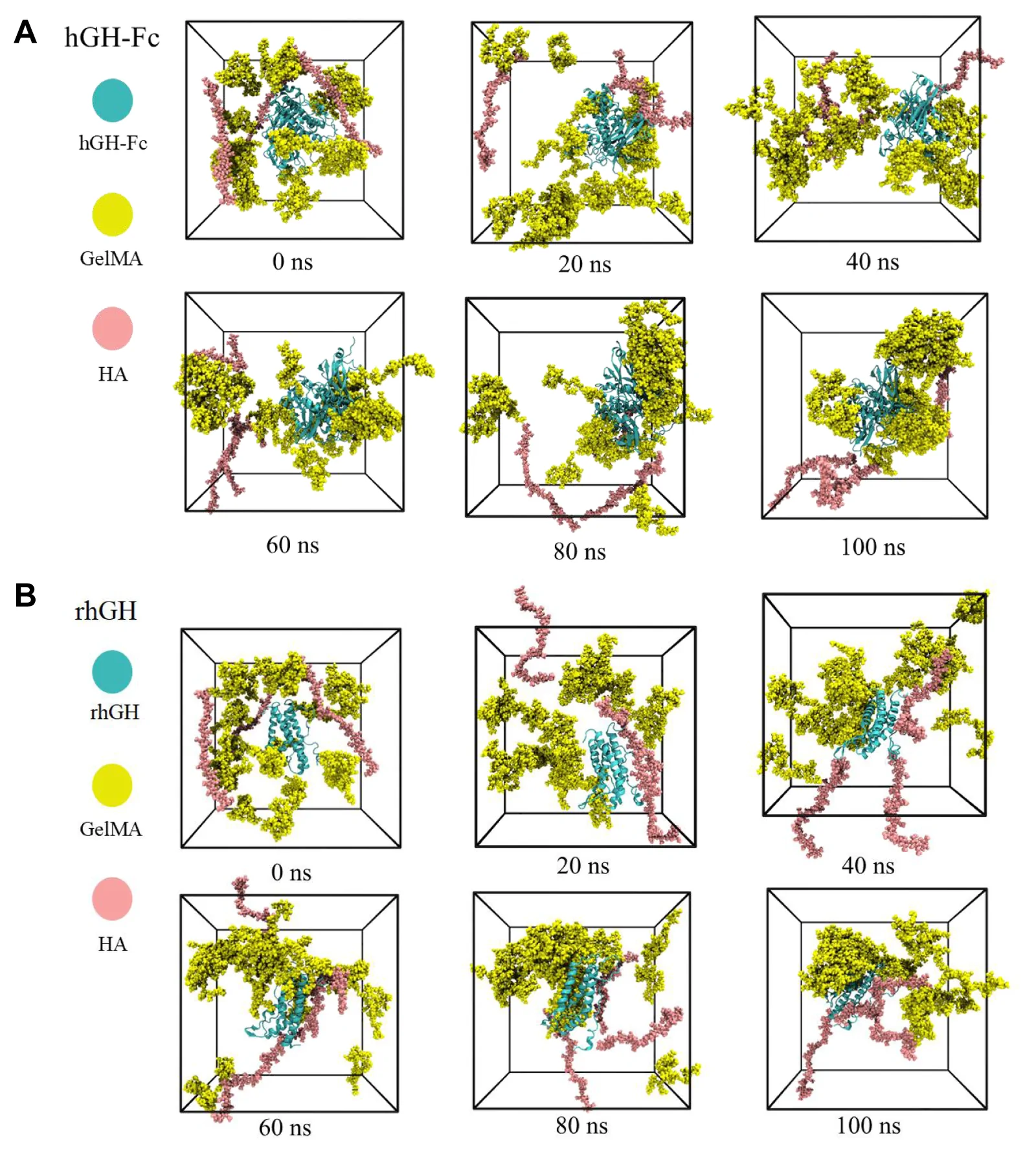
Spatial distribution of hGH-Fc and rhGH in composite matrix. (**A**) Equilibrium conformational distribution of hGH-Fc composite system; (**B**) equilibrium conformational distribution of rhGH composite system.

Molecular dynamics simulation results revealed distinct spatial distribution and interfacial assembly behaviors of rhGH and hGH-Fc in the GelMA/HA composite system (Figure 1). For hGH-Fc, GelMA chains gradually migrated and rearranged around the protein to form a continuous yet loose encapsulating network. HA was mainly distributed at the network periphery with limited direct contact with hGH-Fc. Accordingly, hGH-Fc was embedded in a uniform, low-confinement three-dimensional polymer environment and maintained considerable spatial conformational freedom. In contrast, the rhGH system exhibited obvious local aggregation and asymmetric distribution. GelMA chains rapidly accumulated on the local surface of rhGH to form dense polymer aggregates, while HA chains fully adhered to the protein surface and participated in interfacial binding. Different from the uniform encapsulation of hGH-Fc, rhGH was stably confined within local dense polymer regions, forming an asymmetric system structure. Comparative analysis demonstrated fundamental differences in protein–matrix interaction modes. hGH-Fc interacted weakly and uniformly with GelMA-dominated polymers with negligible HA participation. In comparison, rhGH effectively induced directional densification of the composite network and formed tight interfacial binding with both GelMA and HA, resulting in stronger spatial confinement and more stable composite interfaces.

#### 2.1.2. Protein Conformation and Residue Flexibility

**Figure 2:**
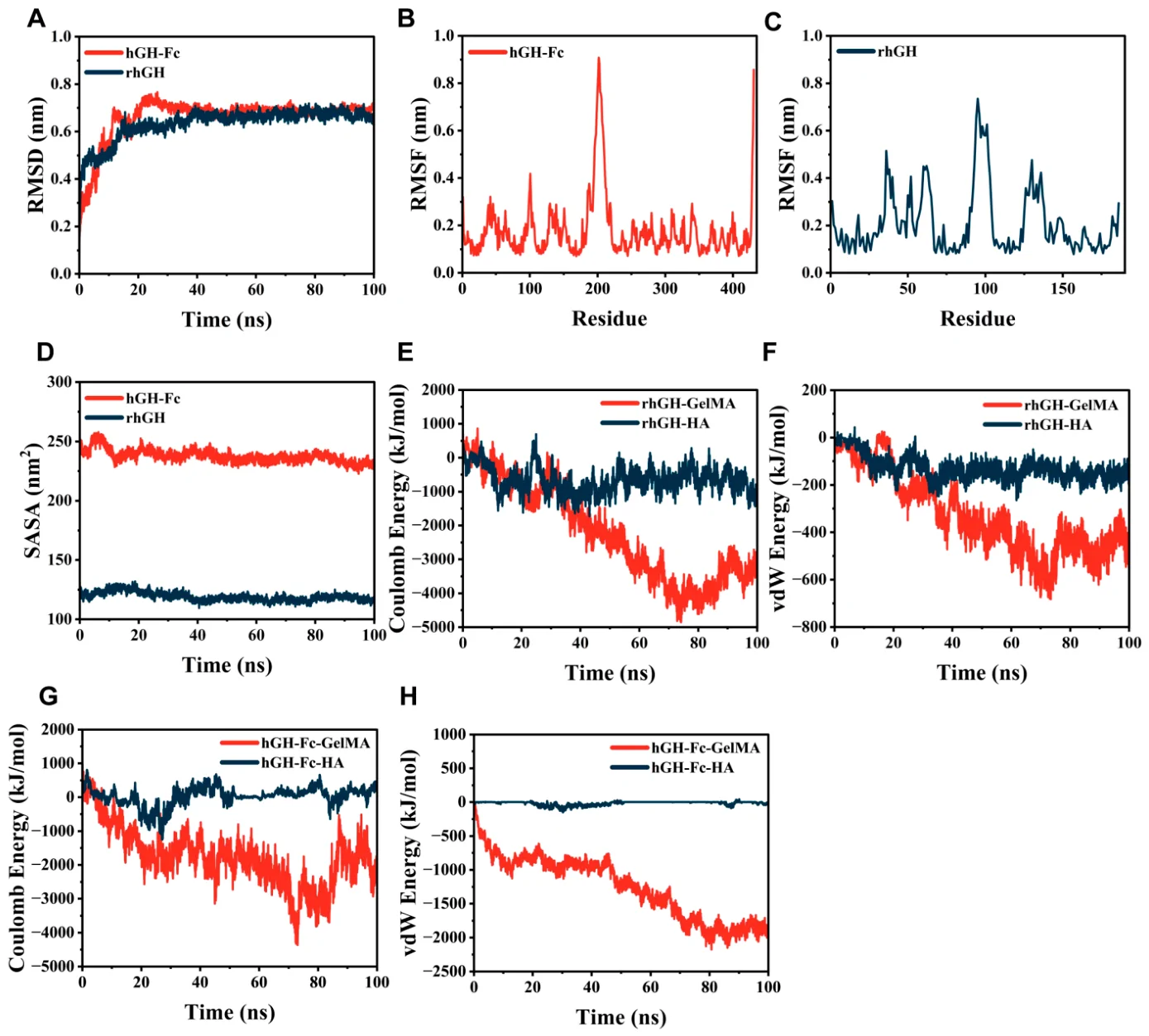
Conformational stability and residue flexibility of hGH-Fc and rhGH. (**A**) RMSD curves of two proteins during 100 ns simulation; (**B**,**C**) RMSF distribution of residue sites of hGH-Fc and rhGH; (**D**) SASA variation in two proteins; (**E**–**H**) non-bonded interaction energy between proteins and GelMA/HA matrix.

RMSD was utilized to evaluate the overall conformational deviation of proteins from the initial structure and judge the equilibrium state of the simulation system (Figure 2A). Both proteins underwent rapid structural adjustment in the early simulation stage to adapt to the GelMA/HA composite environment. The hGH-Fc group required 15–20 ns to reach conformational equilibrium, with a stable RMSD value of 0.70 ± 0.04 nm. In comparison, rhGH achieved equilibrium earlier at 10–15 ns, maintaining a stable RMSD of 0.66 ± 0.03 nm with minor later fluctuations. Statistical analysis (Student’s *t*-test) indicated a statistically significant difference between the two proteins (*p* < 0.05).

RMSF was further adopted to characterize the local residue flexibility of the two proteins at the microscopic level (Figure 2B,C). Most residues of both proteins maintained low fluctuation (0.1–0.2 nm), proving that the protein backbones remained structurally stable throughout the simulation. Nevertheless, hGH-Fc exhibited discretely distributed flexible regions with a prominent maximum fluctuation peak close to 0.9 nm, reflecting uneven local structural flexibility. In contrast, the high-fluctuation regions of rhGH were more concentrated and possessed a lower maximum fluctuation value, endowing rhGH with a more stable structural core and stronger overall structural compactness.

#### 2.1.3. Surface Properties and Intermolecular Interaction

SASA was adopted to evaluate protein conformational compactness and surface hydrophobic exposure, reflecting the encapsulation and shielding effect of the polymer matrix on proteins (Figure 2D). hGH-Fc maintained a high and stable SASA value of 230–250 nm^2^ throughout the simulation, with only minor early fluctuations and a slight declining trend in the late stage. This indicated that hGH-Fc merely underwent subtle local structural adjustments, with GelMA chain coverage mildly reducing its solvent exposure without causing structural unfolding. In contrast, rhGH exhibited a much lower SASA range of 115–125 nm^2^. It achieved structural stability after 30–40 ns of slight early fluctuation, retaining a compact and stable conformation. The stable low SASA of rhGH confirmed its superior structural stability, which was barely disturbed by the surrounding GelMA and HA polymers.

Interaction energy was further analyzed to quantify the non-bonded interaction modes between proteins and the composite matrix, including van der Waals and electrostatic interactions (Figure 2E–H). The interaction of hGH-Fc with the matrix was predominantly GelMA-derived van der Waals interactions, which gradually strengthened and finally stabilized at approximately −2000 kJ/mol, demonstrating continuous tight hydrophobic contact. Meanwhile, hGH-Fc showed negligible interaction with HA, indicating that HA hardly participated in interfacial binding. Conversely, rhGH presented a dual interaction mechanism with both matrix components. It formed moderate van der Waals contact with GelMA and weak binding with HA, while electrostatic interactions served as the dominant driving force for interfacial adhesion. These distinct interaction mechanisms verified that hGH-Fc relied on hydrophobic matching for matrix binding, whereas rhGH was more sensitive to charged HA and tended to form electrostatic-driven stable binding.

#### 2.1.4. Protein–Matrix Contact State

**Figure 3:**
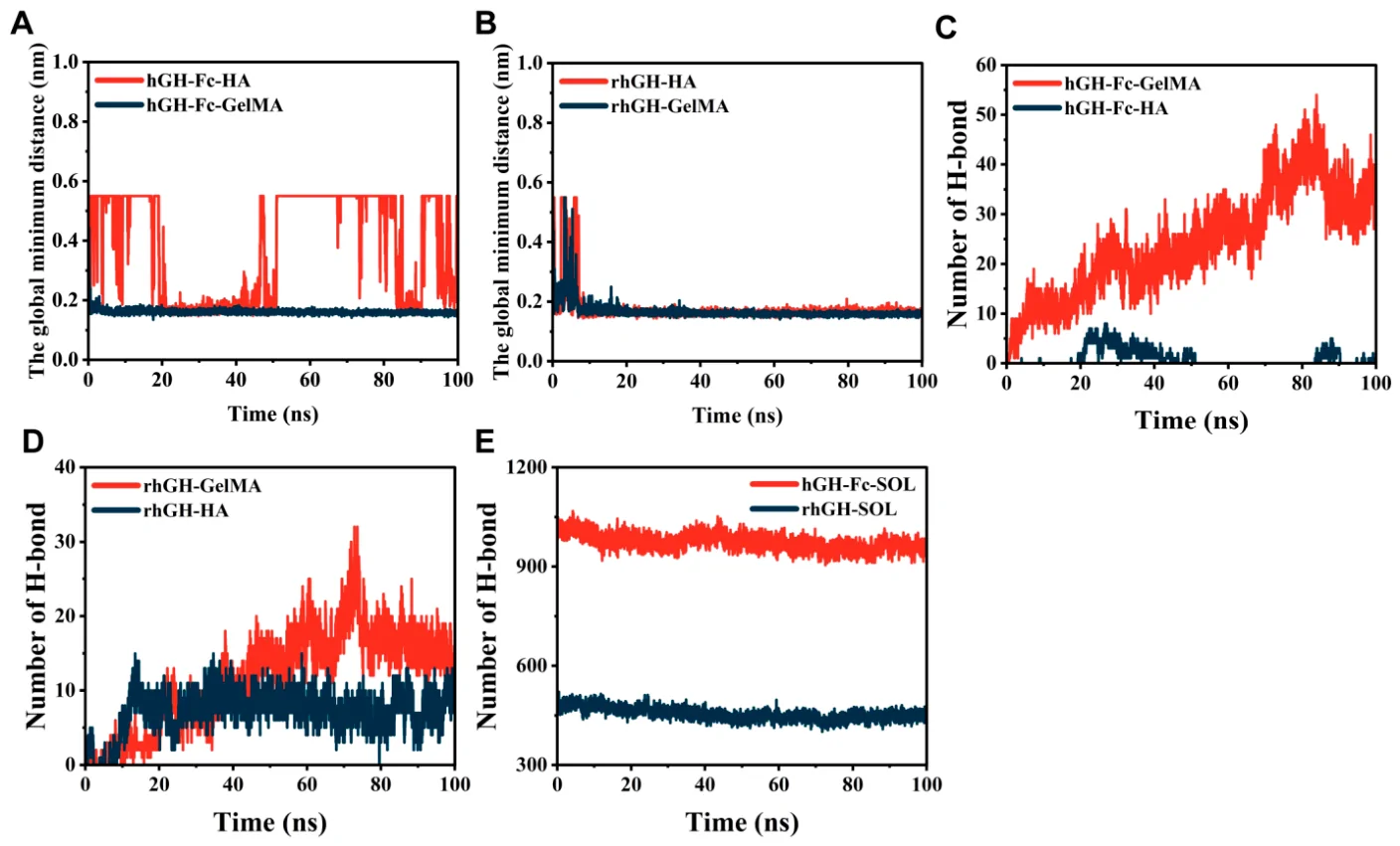
Interfacial contact and hydrogen bonding of protein–matrix systems. (**A**,**B**) Global minimum distance between hGH-Fc/rhGH and GelMA/HA components; (**C**–**E**) time-dependent variation in hydrogen bond numbers between proteins and the matrix.

The global minimum distance was analyzed to characterize the real-time contact state between proteins and the composite matrix (Figure 3A,B). hGH-Fc only maintained stable and close interfacial contact with GelMA throughout the simulation, and almost no effective binding distance was formed with HA, indicating that HA could not participate in the interfacial interaction of hGH-Fc. In contrast, rhGH achieved stable adsorption and tight contact with both GelMA and HA after 10 ns simulation. The stable binding distance confirmed that rhGH could simultaneously interact with the two matrix components, forming a multi-source interfacial contact structure different from hGH-Fc.

#### 2.1.5. Interfacial Hydrogen Bonding Behavior

**Figure 4:**
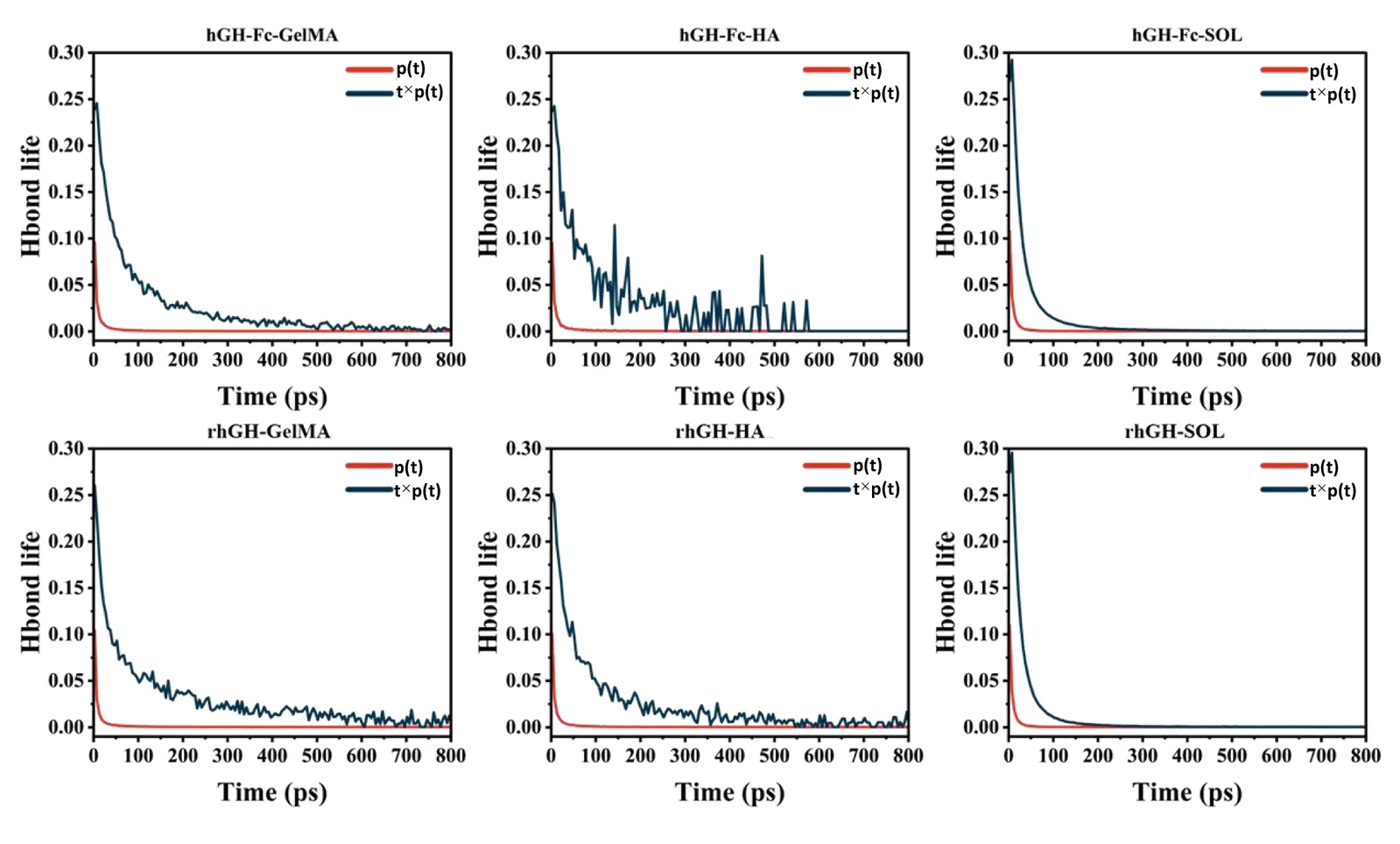
Hydrogen bond lifetime of protein–matrix interaction systems.

Hydrogen bond number and lifetime statistics further verified the differences in interfacial binding stability between the two proteins and the matrix, where the hydrogen bond number was illustrated in (Figure 3C–E) and hydrogen bond lifetime was presented in (Figure 4). rhGH could form abundant and long-lived hydrogen bonds with HA, which provided a stable non-covalent binding force for interfacial adhesion and effectively strengthened the interaction between protein and the composite hydrogel. However, hGH-Fc hardly formed stable hydrogen bonds with HA, and the hydrogen bond interaction was extremely weak and transient. This difference indicated that hydrogen bonding was a key factor for rhGH to achieve tight binding with HA, while it had negligible contribution to the hGH-Fc matrix system.

#### 2.1.6. Molecular Distribution Characteristics

**Figure 5:**
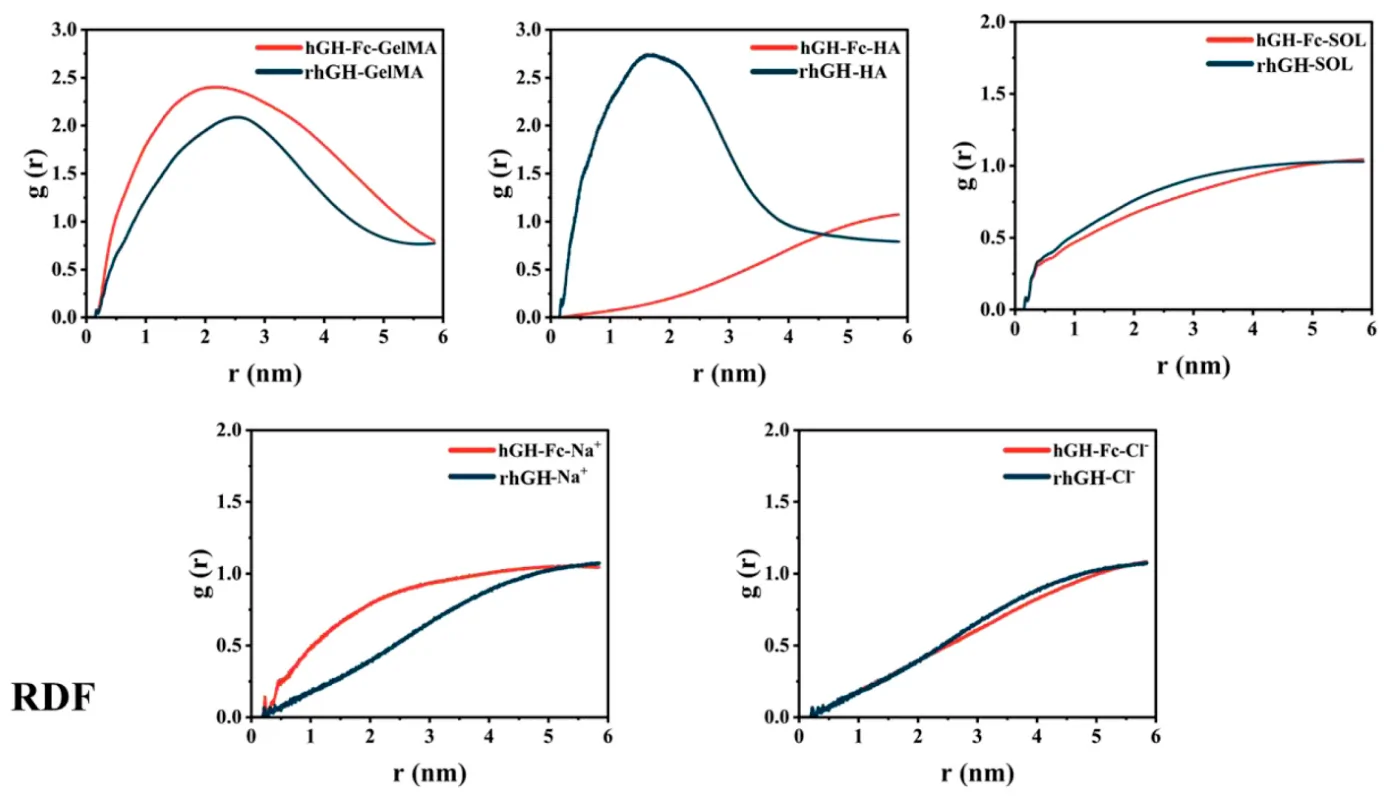
HA radial distribution around hGH-Fc and rhGH.

RDF analysis was performed to reflect the molecular distribution density of matrix components around proteins (Figure 5). The results showed that HA molecules could form an obvious enrichment layer around rhGH, presenting a regular radial distribution state, which further confirmed the effective adsorption and accumulation of HA on the rhGH surface. Nevertheless, HA was randomly and disorderly distributed around hGH-Fc without obvious enrichment behavior. This distinct distribution feature fully explained the weak interfacial interaction and insufficient contact between hGH-Fc and HA at the molecular level.

#### 2.1.7. Protein Secondary Structure Stability

**Figure 6:**
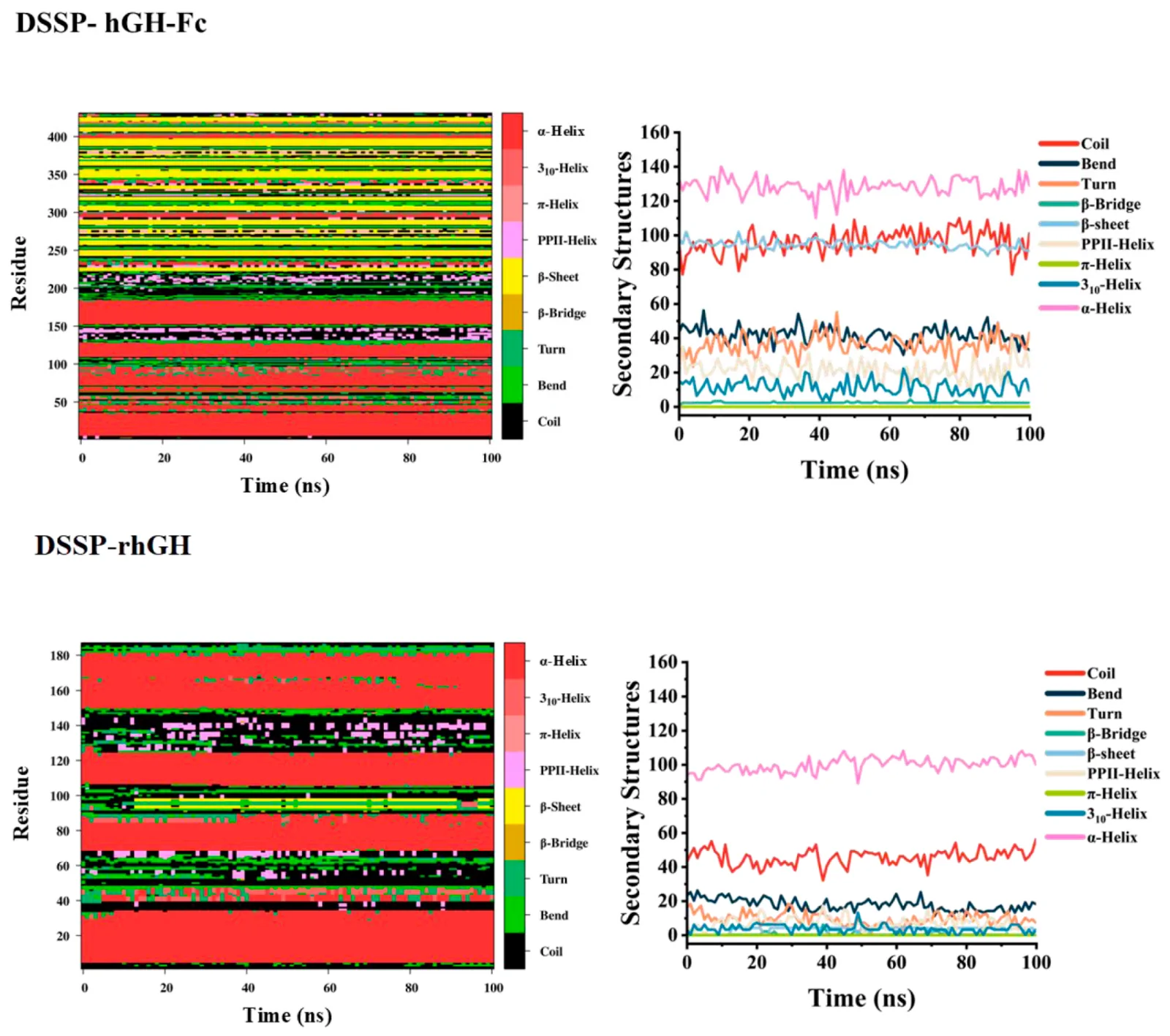
Secondary structure stability of two growth hormone proteins.

DSSP analysis was used to evaluate the secondary structure stability of proteins in the hydrogel environment (Figure 6). Both hGH-Fc and rhGH were dominated by stable α-helix structures during the simulation, without structural dissociation or protein denaturation, proving that the GelMA/HA composite environment had good biocompatibility and could maintain the structural integrity of protein drugs. In addition, rhGH exhibited slightly higher local structural flexibility than hGH-Fc, which was consistent with its local aggregation and tight interfacial binding characteristics.

### 2.2. Microneedle Preparation and Formulation Optimization Results

#### 2.2.1. Optical Morphology Observation

Optical microscopy was used to observe the macroscopic morphology, array regularity, and tip sharpness of microneedles with different GelMA and HA ratios (Figures S1 and S2). Significant morphological differences were observed among groups with varied GelMA concentrations. The 5% GelMA microneedles exhibited short, stout structures with rounded and collapsed tips due to the sparse crosslinked network and insufficient mechanical support of low-concentration GelMA. Increasing HA concentration further deteriorated their structural stability and tip sharpness. The 10% GelMA group achieved optimal molding performance; microneedles with 0.5–2% HA possessed neat arrays, upright bodies, and sharp tips, while excessive 5% HA caused microneedle swelling and tip blunting. For the 15% GelMA group, high crosslinking density endowed microneedles with excellent structural stability but induced stout and blunt features, which became more obvious with elevated HA concentrations. Overall, 10% GelMA combined with 0.5–2% HA was screened as the optimal formulation, balancing fine microscopic morphology and the structural stability of microneedles.

#### 2.2.2. Mechanical Performance Test

**Figure 7:**
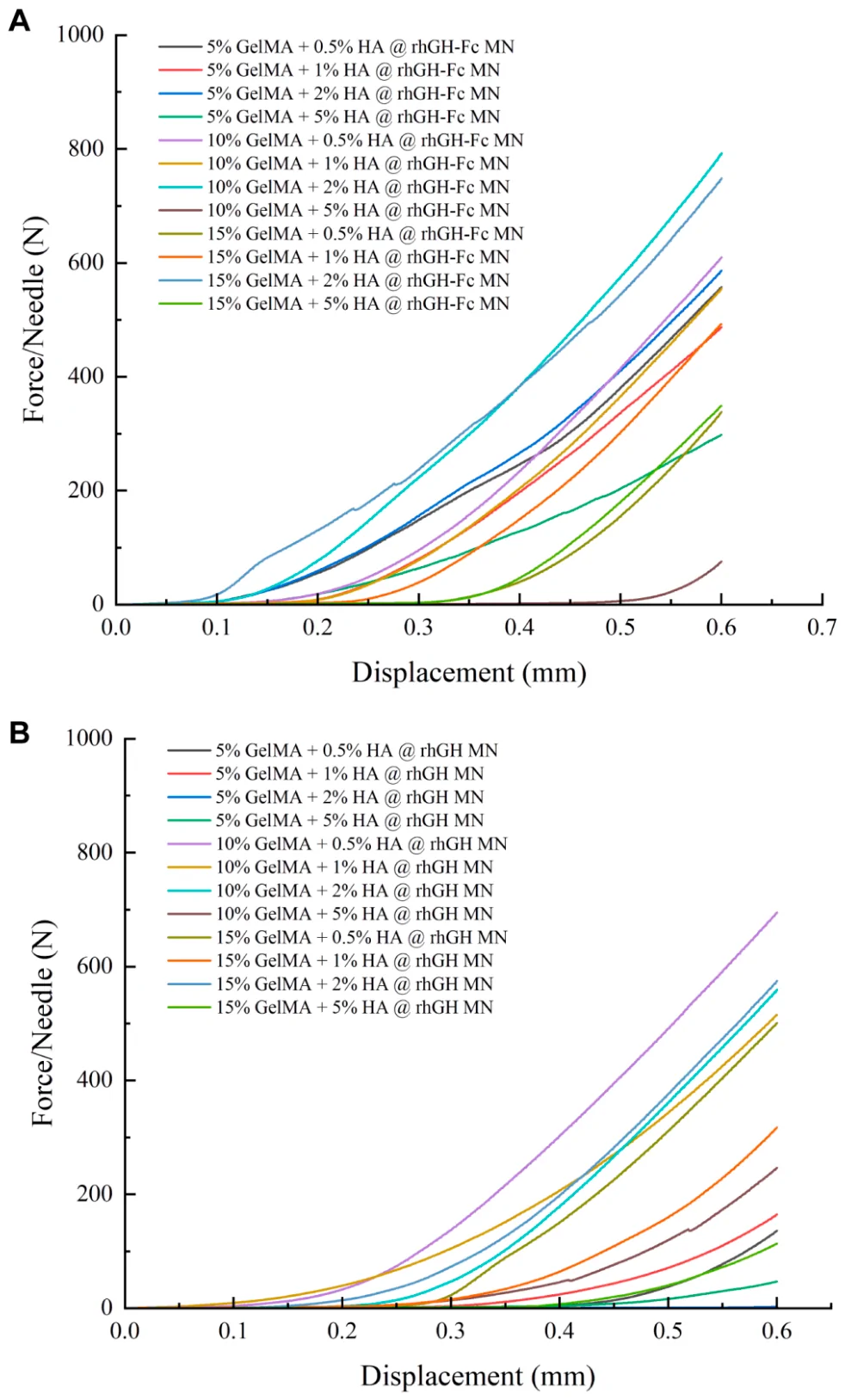
Mechanical compression properties of drug-loaded composite microneedles. (**A**) Force–displacement curves of hGH-Fc-loaded microneedles; (**B**) force–displacement curves of rhGH-loaded microneedles.

Mechanical compression tests were performed to evaluate microneedle compressive stiffness and bearing capacity, with hGH-Fc microneedle data displayed in (Figure 7A, Table 1) and rhGH microneedle properties shown in (Figure 7B, Table 2). GelMA concentration dominated microneedle mechanical rigidity, while HA exerted a secondary weakening effect. For hGH-Fc-loaded microneedles, 5% GelMA had sparse crosslinking and insufficient strength for skin penetration; 10% GelMA achieved stable compressive strength; and 15% GelMA formed dense rigid networks with increased brittleness. rhGH-loaded microneedles presented consistent mechanical trends but slightly lower bearing capacity under identical formulations. Elevated HA concentrations reduced microneedle stiffness by diluting GelMA crosslink density, and all groups exhibited typical compression threshold characteristics. Optimized 10% GelMA + 0.5–2% HA microneedles of both types had a single-tip force above 1.40 N, far exceeding the 0.08 N penetration threshold with balanced strength and flexibility. In contrast, 5% GelMA + 5% HA microneedles showed poor mechanical performance and limited application value.

#### 2.2.3. SEM Microstructure Observation

**Figure 8:**
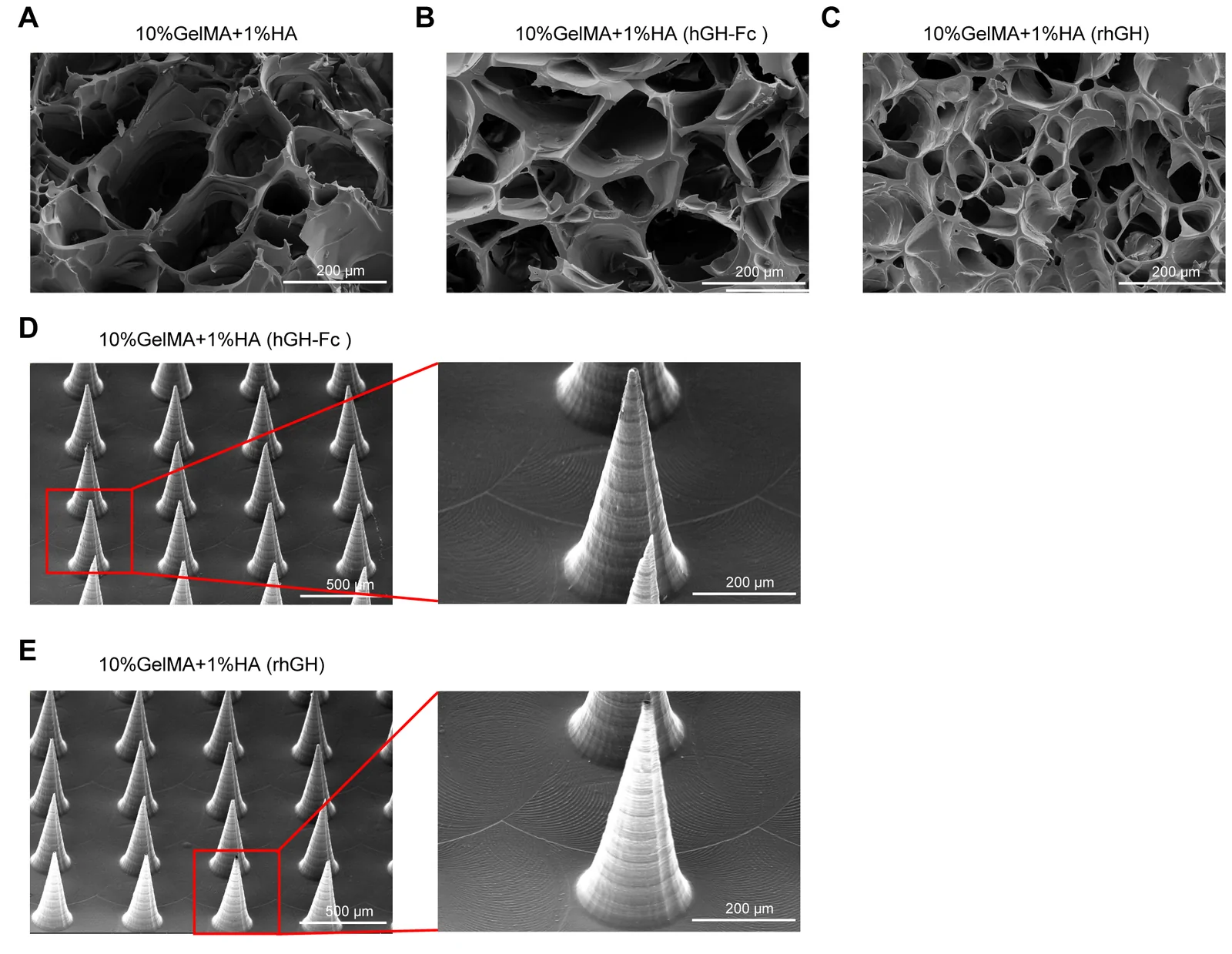
Microstructure and macroscopic morphology of composite microneedles. (**A**) Pore structure of blank GelMA/HA microneedles; (**B**) pore structure of hGH-Fc-loaded microneedles; (**C**) pore structure of rhGH-loaded microneedles; (**D**,**E**) macroscopic array morphology of optimized drug-loaded microneedles. Imaging conditions (all with 0.17 nA, ETD, SE mode): (A) 10 kV, 180×, WD 4.9 mm; (B) 10 kV, 180×, WD 4.2 mm; (C) 10 kV, 180×, WD 5.7 mm; (D) left: 15 kV, 60×, WD 5.8 mm; right: 15 kV, 181×, WD 5.8 mm; (E) left: 15 kV, 60×, WD 5.3 mm; right: 15 kV, 169×, WD 6.0 mm.

Based on the optimal morphological and mechanical properties, 10% GelMA + 1% HA was selected to fabricate blank, hGH-Fc-loaded, and rhGH-loaded microneedle patches with a uniform size of 15 mm × 15 mm. The internal porous microstructure of different microneedle groups was characterized by SEM (Figure 8A–C). Blank GelMA/HA microneedles presented a connected macroporous network with 50–200 μm pores, thin walls, and local structural collapse. Loading hGH-Fc and rhGH significantly optimized the pore structure. hGH-Fc-loaded microneedles possessed uniform 50–150 μm pores with thick continuous walls, while rhGH-loaded groups showed compact pores of 30–150 μm. The two proteins effectively improved hydrogel network densification and stability, which may potentially benefit drug retention and release behavior. The macroscopic array morphology was further verified by SEM (Figure 8D,E). The optimized patch formed a standard 20 × 20 conical microneedle array with 600 μm needle height and 550 μm spacing. The neat, uniform array structure demonstrates excellent structural integrity and reliable skin penetration potential for transdermal delivery.

### 2.3. Physicochemical and Functional Characterization Results of Drug-Loaded Microneedles

#### 2.3.1. FTIR Spectroscopy Characterization

**Figure 9:**
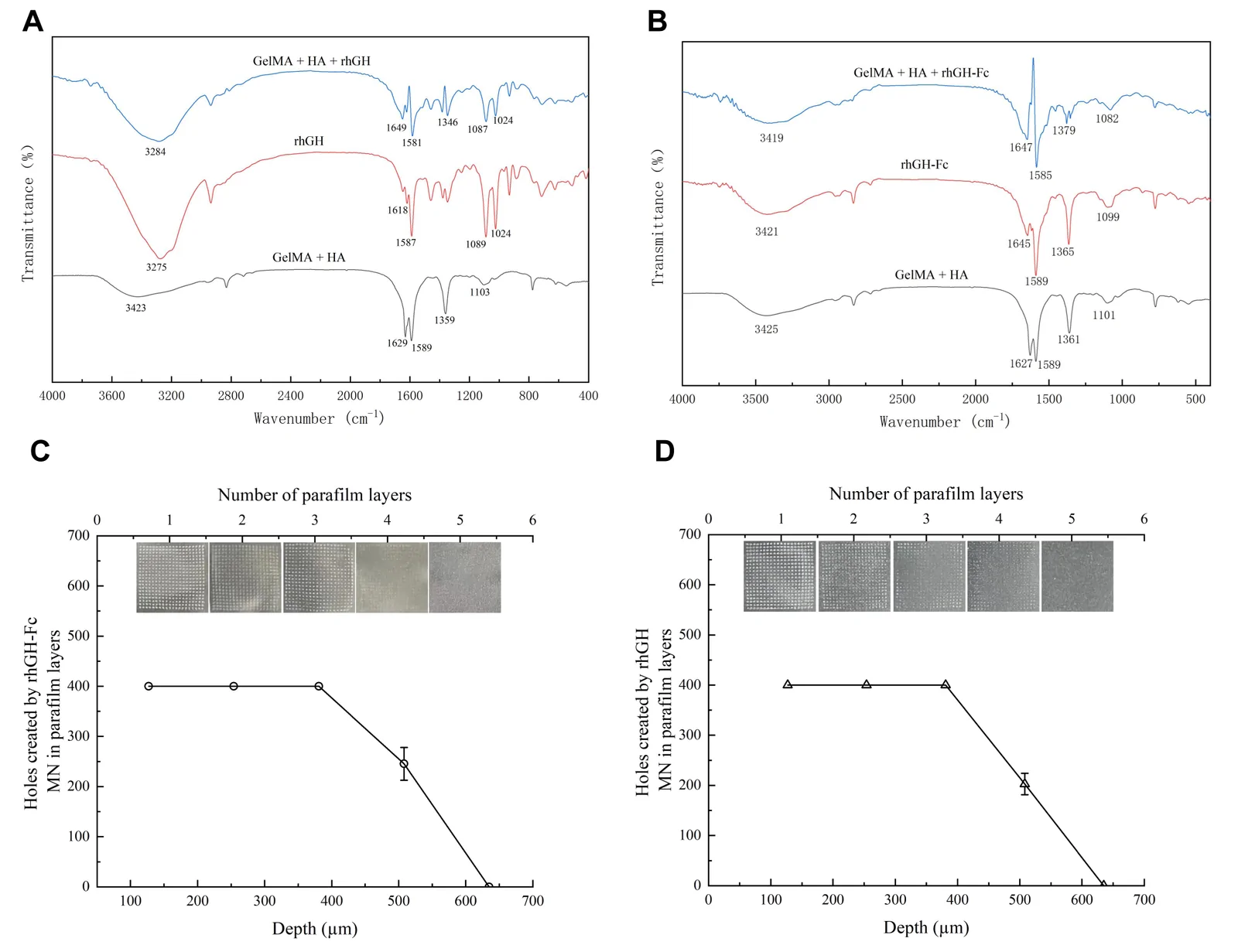
FTIR spectra and percutaneous penetration of drug-loaded microneedles. (**A**) FTIR spectra of blank, pure hGH-Fc, and hGH-Fc-loaded microneedles; (**B**) FTIR spectra of blank, pure rhGH, and rhGH-loaded microneedles; (**C**,**D**) puncture distribution of two drug-loaded microneedles on Parafilm simulated membranes.

FTIR spectroscopy was performed to verify successful drug loading and the structural integrity of proteins in composite microneedles. For long-acting hGH-Fc-loaded microneedles (Figure 9A), slight blue shifts in hydroxyl and amino characteristic peaks at 3200–3500 cm^−1^ suggested the formation of intermolecular hydrogen bonds between hGH-Fc and the GelMA/HA matrix. The intact amide I and amide II bands of hGH-Fc were well retained without significant displacement, indicating that the protein secondary structure was maintained during preparation. For short-acting rhGH-loaded microneedles (Figure 9B), distinct red shifts and enhanced peak intensity in the hydrogen bond region indicated stronger intermolecular interactions between rhGH and the hydrogel network. Similarly, rhGH maintained complete characteristic protein absorption peaks with no new functional group generation. These results fully proved that both proteins were stably loaded via mild physical interactions rather than chemical reactions, achieving good material–protein compatibility.

#### 2.3.2. In Vitro Percutaneous Penetration Performance

The percutaneous penetration performance of optimized microneedles was evaluated using a classical Parafilm M^®^ simulated skin model. Eight layers of 127 μm thick Parafilm M^®^ films were stacked to construct an in vitro penetration model with a total thickness of approximately 1000 μm. After microneedle insertion and removal, puncture pores on each film layer were observed and counted. The penetration results of hGH-Fc-loaded and rhGH-loaded microneedles are presented in (Figure 9C, Table 3) and (Figure 9D, Table 4), respectively. Both microneedle types fully penetrated the first three film layers and partially penetrated the fourth, with no puncture in the fifth layer. hGH-Fc microneedles showed slightly better penetration capacity, and the effective penetration depth of optimized microneedles was calculated to be approximately 500 μm, demonstrating adequate mechanical insertion capability for potential transdermal application.

### 2.4. Discussion

Hydrogel microneedles have become a mainstream minimally invasive transdermal delivery carrier for macromolecular protein drugs, effectively addressing the limitations of traditional subcutaneous injection, including frequent administration and unstable therapeutic effects, which is consistent with the core conclusions of most transdermal delivery studies [16,17,18,19]. Existing studies on GelMA/HA composite microneedles mostly focus on single protein delivery or simple formulation optimization, while few investigations systematically compare the delivery differences between short-acting and long-acting fusion protein drugs. This study fills the research gap regarding molecular mechanism differences between rhGH and hGH-Fc-loaded hydrogel systems, and the comparative research strategy of dual protein loading provides a new reference for the structural design of long/short-acting hormone transdermal delivery carriers.

Molecular dynamics simulation is an effective atomic-level research tool to reveal the interaction mechanism between biomaterials and proteins, which can make up for the limitation that macroscopic characterization cannot explain the internal molecular mechanism of materials. In line with previous studies on protein–hydrogel interfacial interactions, small-molecule active proteins are prone to form multi-site stable non-covalent binding with composite hydrogel matrices, while macromolecular fusion proteins with modified fragments mainly rely on weak hydrophobic interactions for carrier encapsulation [20,21,22]. The Fc fragment of hGH-Fc effectively shields the charged active sites of the GH protein, weakening electrostatic interaction and hydrogen bond formation with HA components, which is the essential reason for the loose encapsulation state of hGH-Fc in the matrix. This molecular-level mechanism interpretation clarifies the internal correlation between protein structure differences and microneedle delivery performance, providing targeted theoretical guidance for the customized preparation of long-acting protein-loaded microneedles.

The formulation screening results of this study verify the universal performance law of GelMA/HA composite hydrogels reported in the previous literature. Moderate HA doping can optimize the porous network structure of the GelMA hydrogel and improve drug loading uniformity and structural flexibility, while excessive HA will reduce the crosslinking density of the composite system and induce structural defects and mechanical attenuation. The optimized 10% GelMA/0.5–2% HA formulation screened in this study achieves the optimal balance of mechanical strength, structural stability, and drug loading performance, which is consistent with the optimal concentration range of composite microneedles for protein delivery in existing studies. FTIR characterization further confirms that physical encapsulation will not cause the chemical modification or denaturation of GH proteins, verifying the good biocompatibility of the composite matrix, which is a necessary prerequisite for the safe delivery of protein macromolecular drugs. However, it should be noted that the structural preservation revealed by FTIR and MD simulations indicates secondary structure integrity rather than direct evidence of preserved biological activity; future functional studies are warranted to confirm bioactivity.

### 2.5. Limitations and Future Perspectives

This study has several limitations common to current research on hydrogel microneedles and protein transdermal delivery. First, the in vitro penetration evaluation only adopts a Parafilm simulated membrane, which lacks the complex physiological microenvironment of biological skin and cannot fully simulate the in vivo transdermal absorption process of drugs [23]. However, the stability of these proteins following microneedle insertion into the acidic skin microenvironment (approximately pH 4.5–5.8) has not been evaluated and therefore remains unknown. Second, the absence of in vitro drug release kinetics data fails to quantitatively confirm the sustained-release advantage of hGH-Fc, which is the core performance index of long-acting drug delivery systems. Third, the storage stability of drug-loaded microneedles under different temperature and humidity conditions is not explored, restricting the industrial transformation and clinical popularization of the preparation. In addition, the ideal aqueous simulation environment cannot simulate the interference of skin interstitial fluid, ions, and other biomolecules, leading to certain discrepancies between simulation results and actual physiological application scenarios [24].

To address these limitations, follow-up work will carry out systematic supplementary experiments. Since the present proof-of-concept study focuses on formulation development, molecular interaction characterization, mechanical evaluation, and preliminary insertion testing, in vitro drug release tests will be performed to quantitatively compare the release behavior of rhGH and hGH-Fc microneedles. Ex vivo skin penetration and in vivo pharmacodynamic studies will be implemented to verify transdermal delivery efficiency and biosafety. Environmental stability tests will also be conducted to optimize storage conditions. Given that such a microneedle platform would likely be classified as a drug–device combination product, comprehensive safety, pharmacokinetic, and quality control data would be prerequisites for clinical translation. Regulatory evaluation would require coordinated assessment of both the drug component (protein stability, bioactivity, and immunogenicity) and the device component (mechanical safety, biocompatibility, and sterility), along with a demonstration of consistent drug content and controlled release characteristics. In addition, the molecular simulation model will be refined by incorporating physiological skin microenvironment factors to further guide formulation development.

## 3. Conclusions

This study fabricated rhGH- and hGH-Fc-loaded GelMA/HA composite microneedles as a potential non-invasive growth hormone delivery platform. The two proteins exhibited distinct matrix binding behaviors: hGH-Fc was loosely encapsulated via hydrophobic van der Waals interactions, whereas rhGH formed stable hydrogen bonds and electrostatic binding with the hydrogel matrix. Orthogonal formulation screening identified the 10% GelMA with 0.5–2% HA as the preferred range, and 1% HA was selected as the representative formulation for detailed characterization. The optimized 10% GelMA + 1% HA formulation possessed good moldability and reliable mechanical performance. The microneedles exhibited mechanical penetration in a simulated skin model. This study elucidates the molecular basis for the differential interactions of short- and long-acting growth hormones with the GelMA/HA matrix and provides a foundation for the further development of growth hormone microneedle formulations.

## 4. Materials and Methods

### 4.1. Materials and Instruments

GelMA (EFL-GM-90), LAP photoinitiator (EFL-LAP), PVA, and HA (EFL-HA-150K) were purchased from Suzhou Yongqinquan Intelligent Equipment, Suzhou, China. Recombinant rhGH and hGH-Fc fusion protein were provided by the Changchun Institute of Biological Products. PBS buffer, potassium bromide, and other analytical reagents were of analytical grade. The main experimental instruments included PDMS microneedle molds, vacuum liquid injection equipment, and a timer-enabled portable desktop curing light (all from Suzhou Yongqinquan Intelligent Equipment, Suzhou, China), as well as an electronic analytical balance (Mettler Toledo, Shanghai, China), optical microscope (Olympus Corporation, Tokyo, Japan), universal mechanical testing machine (Shenzhen Suns Technology, Shenzhen, China), high-vacuum ion sputtering instrument (Quorum Technologies, East Sussex, UK), field emission scanning electron microscope (Hitachi High-Technologies Corporation, Tokyo, Japan), and Fourier transform infrared spectrometer (Thermo Fisher Scientific, Waltham, MA, USA).

### 4.2. Molecular Model Construction and Molecular Dynamics Simulation

The HA molecular model and CHARMM36 force field parameters were constructed using CHARMM-GUI [25,26]. For GelMA, a fragmentation strategy was adopted to generate force field parameters: the gelatin backbone directly adopted the standard parameters of polypeptides in the CHARMM36 force field; the methacrylate side chains were optimized via Gaussian 16, and the corresponding side chain force field parameters were obtained using CGenFF. Subsequently, the side chains were connected to the gelatin backbone through topological editing to form a complete CHARMM36-compatible topology file of the polymer. The structural parameters of rhGH and hGH-Fc were processed via the gmx pdb2gmx module to generate Charmm36 force field files. A 12 nm × 12 nm × 12 nm TIP3P water box was built using Packmol software, where the protein was placed at the center, 20 GelMA molecules and 3 HA molecules were randomly distributed, and the quantity of polymer chains was determined by target concentration and computational feasibility. Counterions were added to a final concentration of 0.15 M to simulate the physiological environment.

All simulation calculations were performed using GROMACS 2025.2 software under periodic boundary conditions [27]. The system was first subjected to 50,000 steps of energy minimization to eliminate steric clashes, followed by 100 ps NVT ensemble equilibration at 310 K to stabilize the system temperature. Subsequently, 100 ps NPT ensemble equilibration was conducted to maintain the system pressure at 1 bar, consistent with the physiological environment. The non-bonded interaction cutoff distance was set to 12 Å, long-range electrostatic interactions were calculated using the PME method, hydrogen bonds were constrained by the LINCS algorithm, and the simulation time step was 2 fs. Finally, a 100 ns production-phase molecular dynamics simulation was carried out. VMD software was used for trajectory visualization, and Gromacs built-in analysis tools were employed to analyze instantaneous conformation, root mean square deviation (RMSD), root mean square fluctuation (RMSF), SASA, interaction energy, global minimum distance, hydrogen bond number and lifetime, radial distribution function (RDF), and Define Secondary Structure of Proteins (DSSP) for protein secondary structure identification [28,29,30,31].

### 4.3. Preparation of GelMA/HA Composite Hydrogel Microneedles

A total of 0.5% LAP photoinitiator solution, 30% GelMA stock solution, and 15% HA stock solution was prepared with PBS buffer and filtered through a 0.22 μm sterile filter. Based on the orthogonal experimental design, GelMA final concentrations were set to 5%, 10%, and 15%, and HA final concentrations were set to 0.5%, 1%, 2%, and 5%. rhGH (5 mg/mL) and hGH-Fc (79.2 mg/mL) stock solutions were separately mixed with different GelMA/HA precursor solutions at a volume ratio of 0.4:1 (drug solution/precursor solution) to prepare drug-loaded microneedle precursor liquids. A 20% PVA solution was prepared as the microneedle substrate material.

The PDMS microneedle mold was sterilized by ultraviolet irradiation, and the precursor solution was injected into the mold under a vacuum pressure of −0.06 to −0.085 MPa and sufficiently degassed. After removing excess solution, the microneedles were pre-cured in situ in the mold with 405 nm ultraviolet light for 60 s. Then, PVA substrate solution was added, and the mold was dried at room temperature under ventilated conditions for 24 h. After complete shaping and demolding, composite hydrogel drug-loaded microneedles with a regular structure and good mechanical properties were successfully fabricated.

### 4.4. Microneedle Characterization and Performance Evaluation

The macroscopic morphology, array arrangement, and tip sharpness of microneedles were observed via an optical microscope. The axial vertical compression test of microneedle arrays was performed at room temperature using a universal mechanical testing machine with a fixed compression rate of 0.04 mm/s to evaluate the mechanical compression properties, and the displacement–force curves were recorded synchronously. Blank and drug-loaded microneedles were vacuum freeze-dried to fix their structures and then subjected to gold-spraying treatment. The microscopic pore structure and needle surface morphology of microneedles were further characterized via SEM.

For Fourier transform infrared spectroscopy characterization, blank microneedles, free rhGH, free hGH-Fc, and drug-loaded microneedles were fully lyophilized to remove residual moisture; then they were ground and mixed uniformly with dry potassium bromide and pressed into tablets. Infrared spectrum scanning was carried out in the wavenumber range of 4000–400 cm^−1^ with appropriate resolution and cumulative scanning times to analyze the functional group changes in materials and intermolecular interactions between components. An eight-layer Parafilm membrane simulated skin model was adopted to evaluate the in vitro penetration performance of microneedles [32].

## Figures and Tables

**Table 1:** Mechanical parameters of hGH-Fc-loaded GelMA/HA composite microneedles with different formulations.

No.	Microneedle Formulation	Maximum Force (N)	Force per Single Tip (N)
1	5% GelMA + 0.5% HA	558.11603	1.40
2	5% GelMA + 1% HA	487.09399	1.22
3	5% GelMA + 2% HA	586.74799	1.47
4	5% GelMA + 5% HA	298.33801	0.75
5	10% GelMA + 0.5% HA	609.77399	1.52
6	10% GelMA + 1% HA	554.80798	1.39
7	10% GelMA + 2% HA	792.80402	1.98
8	10% GelMA + 5% HA	75.864	0.19
9	15% GelMA + 0.5% HA	338.35199	0.85
10	15% GelMA + 1% HA	492.27319	1.23
11	15% GelMA + 2% HA	748.32001	1.87
12	15% GelMA + 5% HA	348.5412	0.87

**Table 2:** Mechanical parameters of rhGH-loaded GelMA/HA composite microneedles with different formulations.

No.	Microneedle Formulation	Maximum Force (N)	Force per Single Tip (N)
1	5% GelMA + 0.5% HA	136.298	0.34
2	5% GelMA + 1% HA	164.73199	0.41
3	5% GelMA + 2% HA	2.714	0.07
4	5% GelMA + 5% HA	47.108	0.12
5	10% GelMA + 0.5% HA	695.14801	1.74
6	10% GelMA + 1% HA	514.76599	1.29
7	10% GelMA + 2% HA	559.47998	1.40
8	10% GelMA + 5% HA	246.85001	0.62
9	15% GelMA + 0.5% HA	501.056	1.25
10	15% GelMA + 1% HA	317.71799	0.79
11	15% GelMA + 2% HA	574.716	1.44
12	15% GelMA + 5% HA	113.662	0.28

**Table 3:** Puncture results of 10% GelMA + 1% HA@hGH-Fc microneedles on Parafilm M^®^ films.

Layer	Number of Puncture Pores			
	Microneedle 1	Microneedle 2	Microneedle 3	Average
1	400	400	400	400
2	400	400	400	400
3	400	400	400	400
4	245	278	213	245
5	0	0	0	0

**Table 4:** Puncture results of 10% GelMA + 1% HA@rhGH microneedles on Parafilm M^®^ films.

Layer	Number of Puncture Pores			
	Microneedle 1	Microneedle 2	Microneedle 3	Average
1	400	400	400	400
2	400	400	400	400
3	400	400	400	400
4	198	226	184	203
5	0	0	0	0

## Data Availability

The data presented in this study are available upon request from the corresponding author.

## Supplementary Materials

The following supporting information can be downloaded at: https://www.mdpi.com/article/10.3390/gels12080666/s1. Figure S1: Optical microscopic characterization of hGH-Fc-loaded GelMA/HA composite microneedles with different formulations; Figure S2: Optical microscopic characterization of rhGH-loaded GelMA/HA composite microneedles with different formulations.
