# Supplementary material for: Molecular Dynamics Simulation and Characterization of GelMA/HA Composite Microneedles for Potential Growth Hormone Transdermal Delivery
Source: Gels. 2026 Jul 24;12(8):666. doi: 10.3390/gels12080666 (PMC13512264; doi:10.3390/gels12080666)
Supplement: Supplementary file 1 [file gels-12-00666-s001.zip › gels-4381140-supplementary.pdf]

Molecular Dynamics Simulation and Characterization of GelMA/HA Composite Microneedles for Potential Growth Hormone Transdermal Delivery

Jianyang Gu, Hao Zhang, Han Liu, Binghuai Peng, Yongbo Qiao and Ping Gong

Supplemental Materials

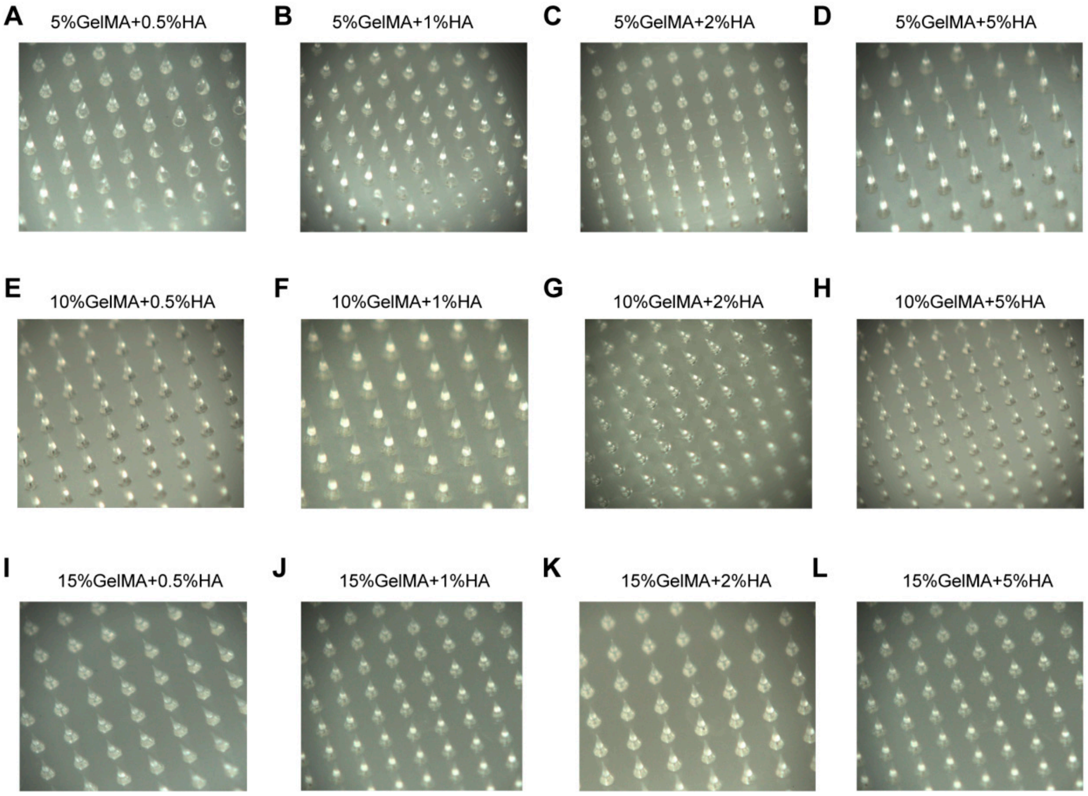

Figure S1. Optical microscopic characterization of hGH-Fc-loaded GelMA/HA composite microneedles with different formulations.

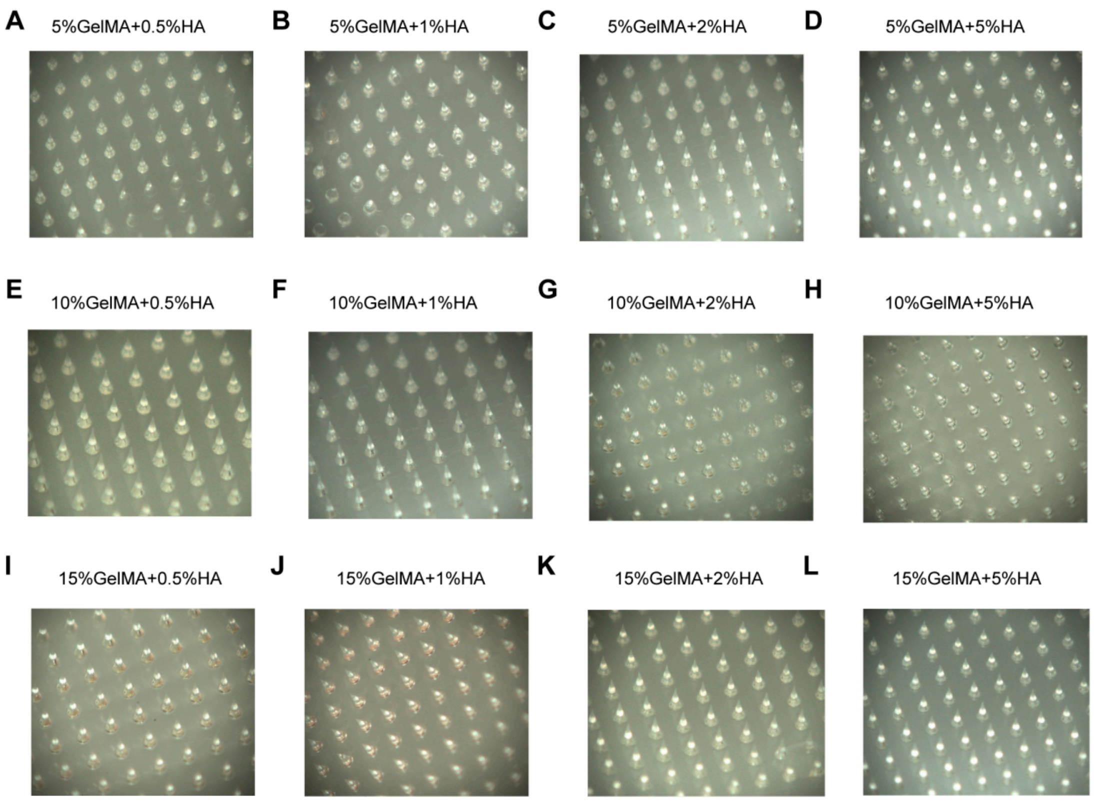

Figure S2. Optical microscopic characterization of rhGH-loaded GelMA/HA composite microneedles with different formulations.
